# Effect of Lacking ZKSCAN3 on Autophagy, Lysosomal Biogenesis and Senescence

**DOI:** 10.3390/ijms24097786

**Published:** 2023-04-24

**Authors:** Xiao-Min Li, Jun-Hao Wen, Ze-Sen Feng, Yun-Shan Wu, Dong-Yi Li, Shan Liang, Dan Wu, Hong-Luan Wu, Shang-Mei Li, Zhen-Nan Ye, Chen Yang, Lin Sun, Ji-Xin Tang, Hua-Feng Liu

**Affiliations:** Guangdong Provincial Key Laboratory of Autophagy and Major Chronic Non-Communicable Diseases, Institute of Nephrology, Affiliated Hospital of Guangdong Medical University, Zhanjiang 524000, China

**Keywords:** ZKSCAN3, autophagy, lysosomal biogenesis, senescence, CRISPR/Cas9, transcriptome sequencing

## Abstract

Transcription factors can affect autophagy activity by promoting or inhibiting the expression of autophagic and lysosomal genes. As a member of the zinc finger family DNA-binding proteins, ZKSCAN3 has been reported to function as a transcriptional repressor of autophagy, silencing of which can induce autophagy and promote lysosomal biogenesis in cancer cells. However, studies in *Zkscan3* knockout mice showed that the deficiency of ZKSCAN3 did not induce autophagy or increase lysosomal biogenesis. In order to further explore the role of ZKSCAN3 in the transcriptional regulation of autophagic genes in human cancer and non-cancer cells, we generated *ZKSCAN3* knockout HK-2 (non-cancer) and Hela (cancer) cells via the CRISPR/Cas9 system and analyzed the differences in gene expression between *ZKSCAN3* deleted cells and non-deleted cells through fluorescence quantitative PCR, western blot and transcriptome sequencing, with special attention to the differences in expression of autophagic and lysosomal genes. We found that ZKSCAN3 may be a cancer-related gene involved in cancer progression, but not an essential transcriptional repressor of autophagic or lysosomal genes, as the lacking of ZKSCAN3 cannot significantly promote the expression of autophagic and lysosomal genes.

## 1. Introduction

Autophagy, an evolutionarily conserved multistep cellular process, plays an essential role in the maintaining of cellular homeostasis via degrading and recycling the damaged organelles and macromolecules within the cell [1]. This process is regulated at different levels from the cell, such as transcriptional regulation and post-transcriptional regulation [2,3]. Recently, regulation of autophagy at the transcriptional level has attracted more and more attention. Many transcription factors, such as TFEB and FoxO3, have been identified to play an essential role in the regulation of autophagic gene transcription [4]. Zinc finger with KRAB and SCAN domains 3 (ZKSCAN3), which belongs to a family of zinc finger transcription factors, is reported to be a master transcriptional repressor of autophagy in human cancer cells [5]. Based on acute knockdown studies of cultured human cancer cells, ZKSCAN3 has been reported to transcriptionally regulate the expression of dozens of genes encoding proteins involved in various steps of autophagy and lysosomal biogenesis [5].

Recently, Pan et al. generated *Zkscan3* knockout mice via the clustered regularly interspaced short palindromic repeats (CRISPR)/CRISPR-associated protein 9 (Cas9) system and found that ZKSCAN3 was not an essential regulator of autophagic or lysosomal gene expression in mice [6]. Why do the results in mice not support the important role of ZKSCAN3 in transcriptional regulation of autophagy? The exact cause of this is unclear, but there are several possible reasons. For example, (1) there are often important differences in cellular responses between acute shRNA knockdown and stable germline knockout; (2) there may be important differences in the role of ZKSCAN3 in humans and mice; (3) there may be significant differences in transcriptional regulation of autophagy between cancer cells and non-cancer cells. 

In order to further understand why the results in human cancer cells are inconsistent with those in mice, and further clarify the role of ZKSCAN3 in the transcriptional regulation of autophagy in human cancer and non-cancer cells, we used the CRISPR/Cas9 system to knock out the *ZKSCAN3* gene in human HK-2 cells (non-cancer cells) and HeLa cells (cancer cells) [7,8], and constructed monoclonal HK-2 cell lines lacking ZKSCAN3 and monoclonal HeLa cell lines lacking ZKSCAN3, respectively. Fluorescence quantitative PCR, western blot and transcriptome sequencing were used to analyze the differences in gene expression between *ZKSCAN3* deleted cells (knockout, KO) and non-deleted cells (wild type, WT), with special attention to the differences in gene expression of autophagy and lysosomal biogenesis. We found that deletion of *ZKSCAN3* had no significant effect on autophagy, lysosomal biogenesis or senescence in eiher HK-2 cells or Hela cells; it is not a master transcriptional repressor of autophagic or lysosomal gene expression, at least in these two cell lines. Based on the results of other studies and our study, we believe that the different results, that knockdown of ZKSCAN3 promotes autophagy and lysosomal biogenesis, while knockout of ZKSCAN3 has no effect on autophagy or lysosomal biogenesis, may be caused by two different ways of gene interventions.

## 2. Results

### 2.1. Generation of ZKSCAN3 KO HK-2 and HeLa Cell Lines via CRISPR/Cas9 System

Human *ZKSCAN3* locates in chromosome 6 (6p22.1) and has three variants. Variant 1 is the longest transcript, which encodes the longer isoform (isoform 1). Variant 1 and variant 2 encode the same protein, whereas variant 3 differs in the 5’UTR, which lacks a part of the 5’ coding region, and initiates translation at a downstream start codon. The resulting protein (isoform 2) coded by variant 3 has a distinct N-terminus and is shorter than isoform 1. To further explore the function of ZKSCAN3 in the regulation of autophagy and lysosomal biogenesis, we generated *ZKSCAN3*-KO HK-2 and *ZKSCAN3*-KO HeLa cell lines via causing a frameshift mutation by deleting several nucleotides in the third exon region through the CRISPR/Cas9 system [9,10].

**Figure 1:**
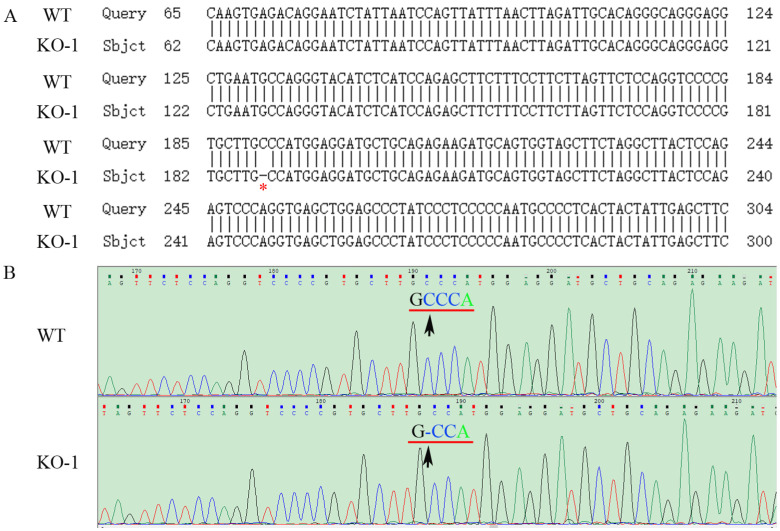
Genome PCR product sequencing of the WT and the mutated *ZKSCAN3* (KO-1) in HK-2 cells. (**A**) The result of partial sequence alignment of *ZKSCAN3* WT and KO genomes. The site marked by the red asterisk is the site of the nucleotide deletion (one C missing). (**B**) The WT and KO partial *ZKSCAN3* gene sequencing trace. The black arrow points to the nucleotide deletion site. KO-1: One nucleotide is missing.

**Figure 2:**
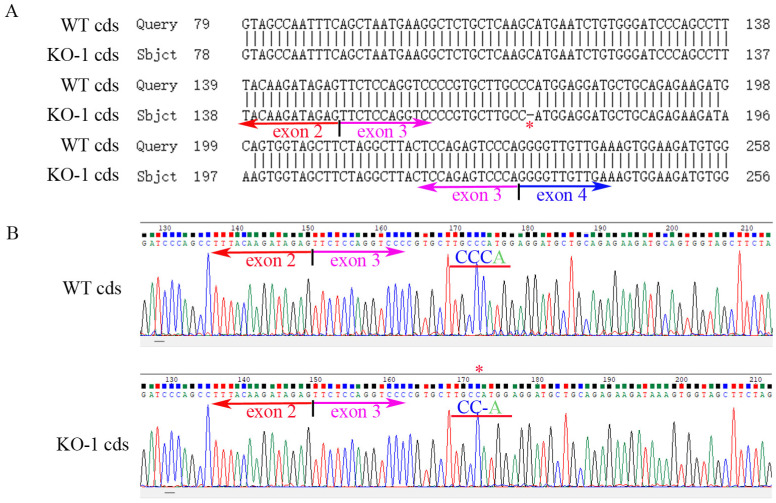
Partial *ZKSCAN3* cDNA PCR product sequencing of the WT and the mutated (KO-1) HK-2 cells. (**A**) The result of partial sequence alignment of *ZKSCAN3* WT and KO coding sequence (cds). The site marked by the red asterisk is the site of the nucleotide deletion (one C missing). (**B**) The WT and KO partial *ZKSCAN3* cDNA sequencing trace. KO-1: One nucleotide is missing. cds: coding sequence.

**Figure 3:**
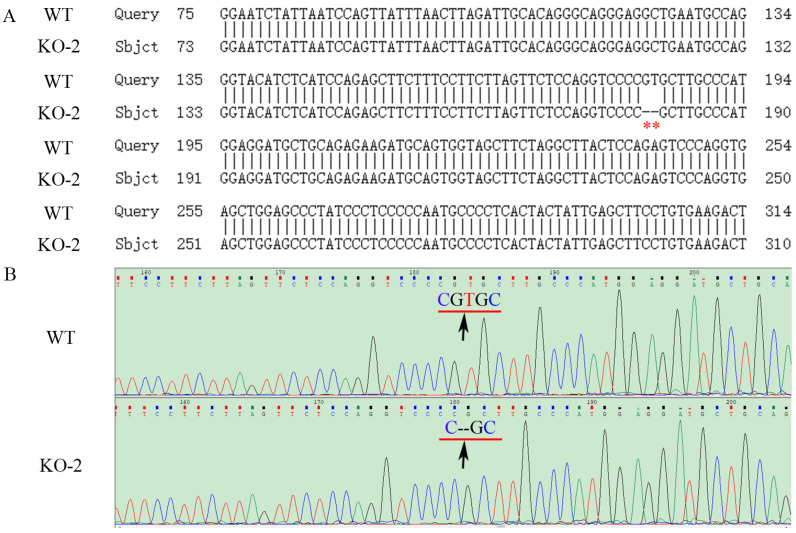
Genome PCR product sequencing of the WT and mutated *ZKSCAN3* (KO-2) in Hela cells. (**A**) The result of partial sequence alignment of *ZKSCAN3* WT and KO genomes. The site marked by the red asterisk is the site of the nucleotide deletion (one G and one T missing). (**B**) The WT and KO partial *ZKSCAN3* gene sequencing trace. The black arrow points to the nucleotide deletion site. KO-2: Two nucleotides are missing.

**Figure 4:**
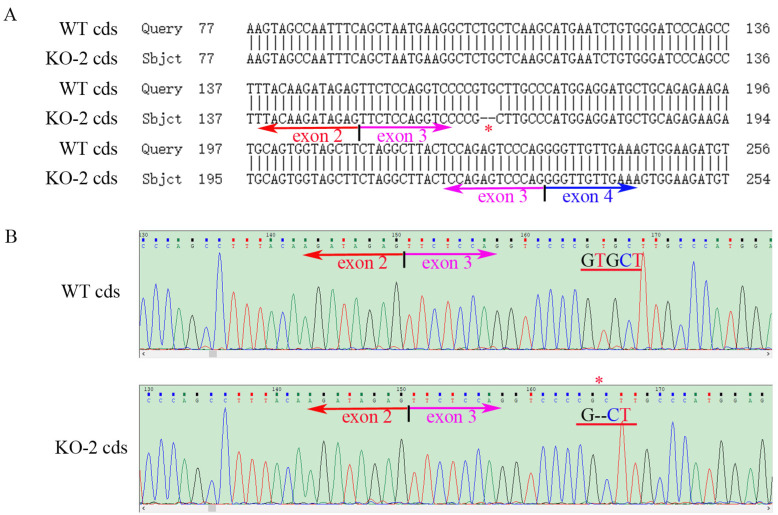
Partial *ZKSCAN3* cDNA PCR product sequencing of the WT and mutated *ZKSCAN3* (KO-2) Hela cells. (**A**) The result of partial sequence alignment of *ZKSCAN3* WT and KO coding sequence (cds). The site marked by the red asterisk is the site of the nucleotide deletion (one G and one T missing). (**B**) The WT and KO partial *ZKSCAN3* cDNA sequencing trace. KO-2: Two nucleotides are missing.

**Figure 5:**
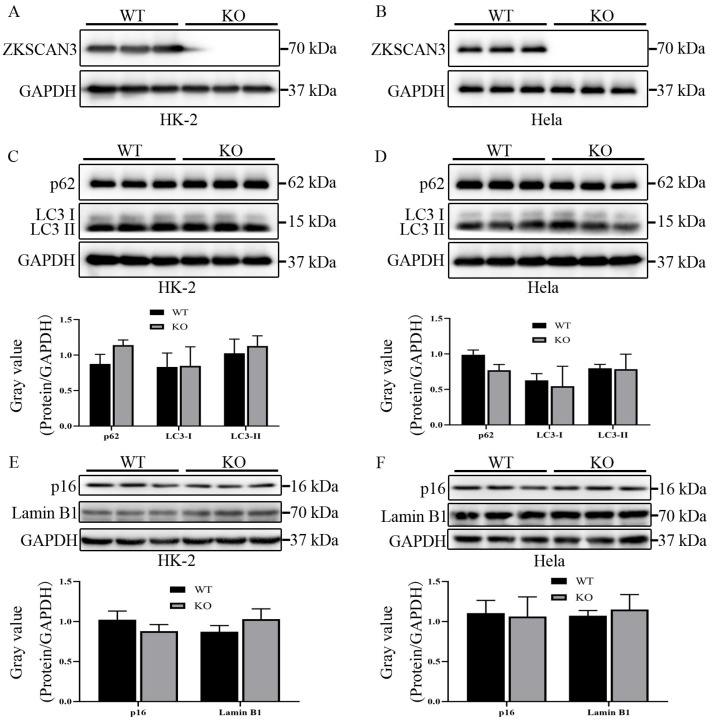
The protein level of ZKSCAN3, p62, LC3I/II, p16 and lamin B1 in WT and *ZKSCAN3* KO HK-2 and Hela cell lines. (**A**) The protein level of ZKSCAN3 in WT and *ZKSCAN3* KO HK-2 cells. (**B**) The protein level of ZKSCAN3 in WT and *ZKSCAN3* KO Hela cells. (**C**) The protein level of p62 and LC3I/II in WT and *ZKSCAN3* KO HK-2 cells. (**D**) The protein level of p62 and LC3I/II in WT and *ZKSCAN3* KO Hela cells. (**E**) The protein level of p16 and lamin B1 in WT and *ZKSCAN3* KO HK-2 cells. (**F**) The protein level of p16 and lamin B1 in WT and *ZKSCAN3* KO HK-2 cells. GAPDH as internal reference. Each band represents a replicate, each group (WT or KO) has three biological replicates.

The constructed plasmids (PX458-*ZKSCAN3*-gRNA) were transfected into HK-2 and Hela cells by liposomes, and positive cells were screened by the single cell selection techniques, and the selected single cell was cultured in a 24-well plate (Figures S1 and S2). The number of cells was expanded by passage, and then the cells were collected to extract protein, total RNAs and genome, and the genotype of *ZKSCAN3* mutants was determined by western blot, PCR and sequencing. We obtained two mutants of *ZKSCAN3* using the CRISPR/Cas9 system, one in HK-2 cells by deleting 1 nucleotide and the other in Hela cells lacking 2 nucleotides. By amplifying and sequencing the genome sequences and cDNA sequences, we have confirmed these mutants at the genomic level and transcriptional level (Figure 1, Figure 2, Figure 3 and Figure 4). To further test whether these two mutants could lead to the complete losing of ZKSCAN3, we performed western blot, and found that ZKSCAN3 cannot be detected in either mutated cell line via western blot (Figure 5A,B). These results indicate that we have successfully constructed *ZKSCAN3*-KO HK-2 and Hela cell lines through the CRISPR/Cas9-mediated genome editing system.

### 2.2. Deletion of ZKSCAN3 Has No Significant Effects on the Regulation of Autophagy and Senescence in HK-2 and HeLa Cells

To explore the effect of *ZKSCAN3* KO on the autophagic degradation in HK-2 and Hela cells, we detected the LC3-II/I protein level and p62 protein level in WT and ZKSCAN3 KO cells. ATG8/LC3, a lipid-conjugated ubiquitin-like protein, is required for the formation of autophagosomes [11,12]. During the formation of autophagosome, the soluble LC3-I is conjugated to phosphatidyl ethanolamine by the cysteine protease ATG4B, which allowed for tethering of the resultant LC3-II to autophagic vacuoles, an essential step in phagophore closure to generate an autophagosome; therefore, the LC3-II protein level indicates the degradation of autophagy [11]. Sequestosome-1 (SQSTM1)/p62, a selective autophagic receptor, can recruit and deliver ubiquitinated intracellular cargos for degradation via the autophagy-lysosomal pathway [13,14]. As a link between LC3 and ubiquitinated cargo, p62 and its bounded polyubiquitinated cargos can be incorporated into the autophagosome and then degraded by auto lysosomes; therefore, p62 is also believed to be an index of autophagic degradation [11]. We found that the losing of ZKSCAN3 cannot upregulate the protein level of LC3-II or cannotdownregulate the p62 protein level in either HK-2 cells or Hela cells (Figure 5C,D). These results suggest that *ZKSCAN3* KO cannot promote the ability of the autophagic degradation in either HK-2 cells or Hela cells.

Previous studies showed that silencing *ZKSCAN3* by small hairpin RNAs (shRNAs) in bladder cancer cells (UC13) can promote senescence of them [5], which may be due to the degradation of lamin B1 via autophagy, a type of selective autophagy—nuclear autophagy [15,16,17]. Lamin B1, a key component of the nuclear lamina, is associated with transcriptionally inactive heterochromatin domains and plays an essential role in cellular senescence [18,19]. We explored the protein level of lamin B1 and p16 (MTS-1/CDKN2/INK4a), a cyclin-dependent kinase-4 inhibitor that is highly expressed in senescent cells, in WT and ZKSCAN3 KO cells, and found that the protein level of p16 and lamin B1 had no significant difference between WT and ZKSCAN3 KO cells (Figure 5E,F). These observations suggest that ZKSCAN3 KO may be unable to promote the senescence of HK-2 and Hela cells.

### 2.3. ZKSCAN3 KO Cannot Promote the Transcription of Autophagic and Lysosomal Genes in HK-2 and Hela Cells

**Figure 6:**
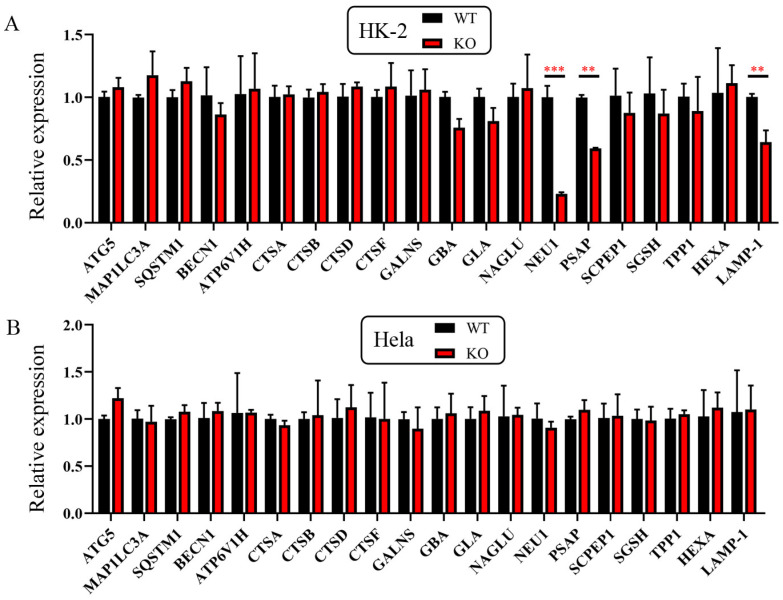
The mRNA level of ATG5, MAP1LC3A, SQSTM1, BECN1, ATP6V1H, CTSA, CTSD, CTSF, GALNS, GBA, GLA, NAGLU, NEU1, PSAP, SCPEP1, SGSH, TPP1, HEXA and LAMP-1 in WT and *ZKSCAN3* KO HK-2 and Hela cell lines. (**A**) The mRNA level of ATG5, MAP1LC3A, SQSTM1, BECN1, ATP6V1H, CTSA, CTSD, CTSF, GALNS, GBA, GLA, NAGLU, NEU1, PSAP, SCPEP1, SGSH, TPP1, HEXA and LAMP-1 in WT and *ZKSCAN3* KO HK-2 cells. (**B**) The mRNA level of ATG5, MAP1LC3A, SQSTM1, BECN1, ATP6V1H, CTSA, CTSD, CTSF, GALNS, GBA, GLA, NAGLU, NEU1, PSAP, SCPEP1, SGSH, TPP1, HEXA and LAMP-1 in WT and *ZKSCAN3* KO HK-2 cells. GAPDH mRNA as internal reference. Each group (WT or *ZKSCAN3* KO) has three biological replicates. KO: *ZKSCAN3* KO. **: *p* < 0.01, ***: *p* < 0.001.

We next sought to explore whether the *ZKSCAN3* KO can promote the transcription of autophagic and lysosomal genes as it is believed that ZKSCAN3 is a transcriptional repressor. We harvested the total mRNA from WT and *ZKSCAN3* KO cells. We examined the expression of autophagic and lysosomal genes by reverse transcription and fluorescence quantitative PCR (qPCR) (Table S1). We found that *ZKSCAN3* KO was unable to promote the expression of a host of autophagic and lysosomal genes that were reported to be direct and indirect targets of ZKSCAN3 (Figure 6). While we did notice that some genes (such as NEU1, PSAP and LAMP-1) were significantly reduced in ZkSCAN3 KO HK-2 cells, this reduction was actually the opposite of what would be expected after the loss of the repressor (Figure 6A). These results indicated that *ZKSCAN3* KO cannot promote the transcription of autophagic and lysosomal genes in either HK-2 cells or Hela cells.

### 2.4. Effects of ZKSCAN3 KO on the Transcriptome of HK-2 and Hela Cells

**Figure 7:**
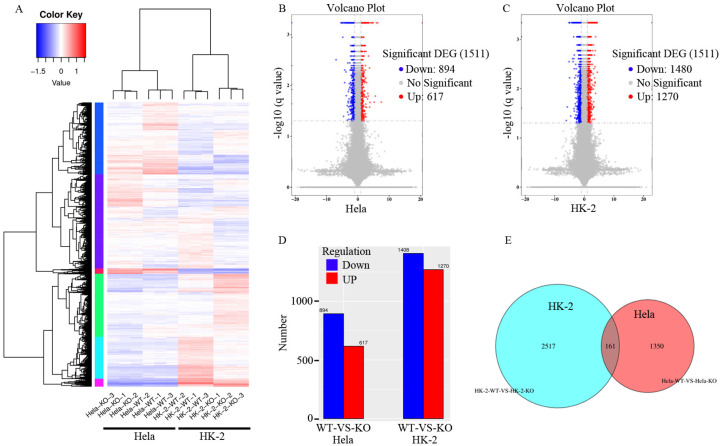
Gene differential expression analysis of WT and *ZKSCAN3* KO HK-2 and Hela cells. (**A**) Cluster map of differential genes in WT and *ZKSCAN3* KO HK-2 and Hela cells. The value log10 (FPKM + 1) was used for clustering, with red representing high-expression genes and blue representing low-expression genes. The color ranges from blue to red, indicating increased gene expression. (**B**) The volcano map of differentially expressed genes in Hela cells. The red dots indicate the upregulated significantly differentially expressed genes and the blue dots indicates the downregulated. The horizontal coordinate represents the change in gene expression fold in different samples. The ordinate represents the statistical significance of the difference in gene expression. (**C**) The volcano map of differentially expressed genes in HK-2 cells. (**D**) The number of upregulated and downregulated significantly differentially expressed genes in Hela and HK-2 cells. (**E**) Venn diagram of significantly differentially expressed genes. The Venn diagram shows the number of the specific significantly differentially expressed genes and shared significantly differentially expressed genes between HK-2 and Hela cells.

To further and comprehensively characterize the transcriptome-wide transcriptional alterations caused by *ZKSCAN3* KO, we performed whole transcriptome sequencing in WT and *ZKSCAN3* KO cells. Whole transcriptome sequencing has been widely used in basic research, clinical diagnosis and drug research and development to obtain the sequence information of almost all transcripts of specific tissues or organs of a certain species in a certain state [20,21,22]. Through the new generation of high-throughput sequencing, we found that *ZKSCAN3* KO in HK-2 cells resulted in 1408 genes being significantly downregulated and 1270 genes significantly upregulated compared with WT HK-2 cells (Table S2). Whereas in Hela cells, *ZKSCAN3* KO led to 894 genes being significantly downregulated and 617 genes significantly upregulated compared with the WT Hela cells (Table S3). Of these significantly differentially expressed genes, 161 were present in both HK-2 and Hela *ZKSCAN3* KO cells (Figure 7).

**Figure 8:**
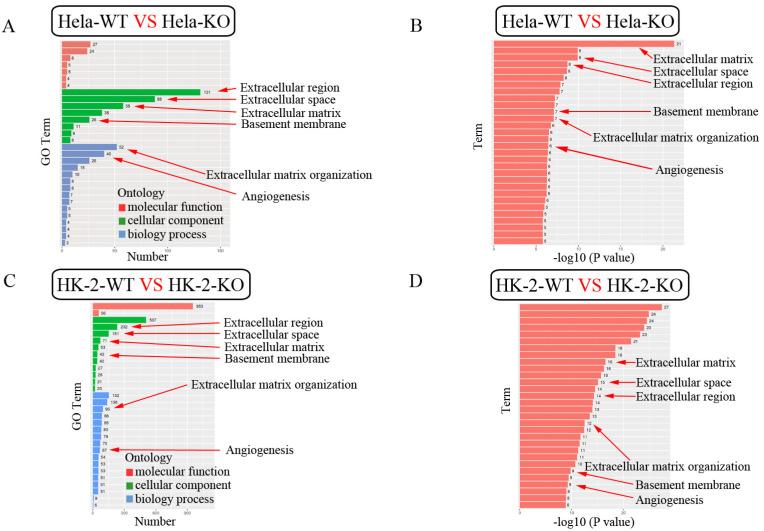
GO enrichment histogram of significantly differentially expressed genes in Hela cells and HK-2 cells. (**A**) GO enrichment histogram in Hela cells, where the ordinate is the enriched GO term, and the abscissa is the number of significantly differentially expressed genes in this term. Different colors are used to distinguish the biological process, cellular component, and molecular function. (**B**) GO enrichment *p* value bar graph in Hela cells, where the ordinate is the enriched GO term and the abscissa is the term-log10 (*p*-value) value. (**C**) GO enrichment histogram in HK-2 cells, where the ordinate is the enriched GO term, and the abscissa is the number of significantly differentially expressed genes in this term. Different colors are used to distinguish the biological process, cellular component, and molecular function. (**D**) GO enrichment *p* value bar graph in HK-2 cells, where the ordinate is the enriched GO term and the abscissa is the term-log10 (*p*-value) value.

**Figure 9:**
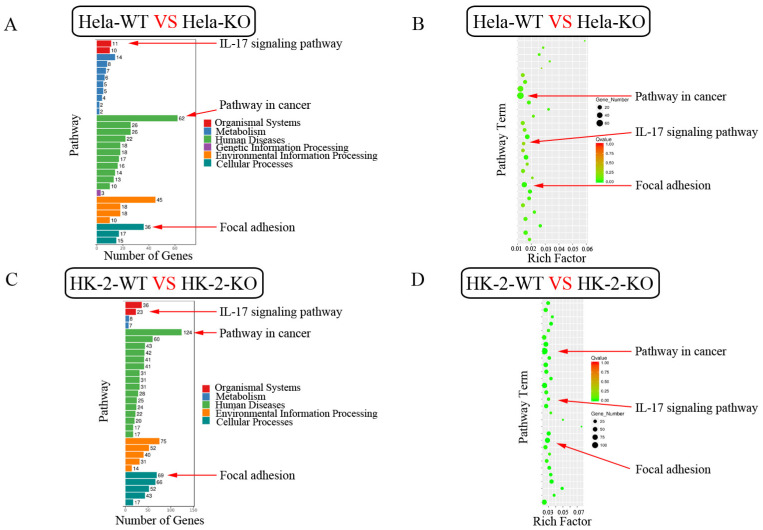
KEGG enrichment map of significantly differentially expressed genes in Hela cells and HK-2 cells. (**A**) KEGG annotated classification bar chart of significantly differentially expressed genes in Hela cells, with the vertical axis indicating pathway names and the horizontal axis indicating the number of genes. (**B**) KEGG enrichment distribution point diagram in Hela cells. Pathway names are represented on the vertical axis. Rich factor is represented on the horizontal axis; the number of significantly differentially expressed genes in this pathway is represented by the size of the dots, and the colors of the dots correspond to different Q value ranges. (**C**) KEGG annotated classification bar chart of significantly differentially expressed genes in HK-2 cells, with the vertical axis indicating pathway names and the horizontal axis indicating the number of genes. (**D**) KEGG enrichment distribution point diagram in HK-2 cells. Pathway names are represented on the vertical axis, rich factor is represented on the horizontal axis; the number of significantly differentially expressed genes in this pathway is represented by the size of the dots, and the colors of the dots correspond to different Q value ranges.

The GO enrichment bar chart directly reflects the number distribution of differential genes in the GO term enriched in the biological process, cellular component and molecular function. We selected 30 GO terms with the most significant enrichment and showed them in Figure 8. Among these GO terms, extracellular region, extracellular space, extracellular matrix, extracellular matrix organization, basement membrane and angiogenesis were found in both HK-2 and Hela cells (Figure 8). In vivo, different genes coordinate with each other to perform their biological functions. Through the significant enrichment of the pathway, the most important biochemical metabolic pathways and signal transduction pathways involved in differentially expressed genes can be identified. KEGG (Kyoto Encyclopedia of Genes and Genomes) is the main public database on the pathway [23]. Pathway significance enrichment analysis using KEGG pathway as unit, hypergeometric test was applied to find out the pathway that showed significant enrichment in differentially expressed genes compared with the whole genome background. The 30 pathway entries with the most significant enrichment were selected and shown in Figure 9. Among these pathways, a pathway in cancer, focal adhesion and IL-17 signaling were found both in HK-2 and Hela cells (Figure 9). These results suggest that ZKSCAN3 is more likely to be involved in cancer and immune regulation than in autophagy and lysosomal biogenesis.

## 3. Discussion

In this study, we generated *ZKSCAN3* KO HK-2 and Hela cells, and explored the effects of *ZKSCAN3* KO on the autophagy, lysosomal biogenesis and senescence through qRT-PCR, western blot and transcriptome sequencing. Our results indicate that *ZKSCAN3* KO was unable to promote the transcription of autophagic and lysosomal genes in either HK-2 cells or Hela cells. Deletion of *ZKSCAN3* did not affect the expression of age-related p16 or lamin B1 in either HK-2 cells or Hela cells. By transcriptome sequencing, we found that the *ZKSCAN3* KO resulted in significantly higher or lower expression of many genes associated with cancer and immunity both in HK-2 and Hela cells, suggesting that ZKSCAN3 may be an essential regulator of cancer progression but not a master transcriptional repressor of autophagic and lysosomal genes.

ZKSCAN3 belongs to a family of transcriptional repressor proteins and has been proved to play an essential role in cancer progression via different ways [24,25,26,27,28,29]. For example, Lee et al. showed that ZKSCAN3 often over-expressed in uterine cervical cancer and correlated with the poor clinical outcome [30]. Moreover, ZKSCAN3 is also overexpressed in hepatocellular carcinoma tissues and promotes hepatocellular carcinoma migration and invasion [31]. Herein, we found that the *ZKSCAN3* KO resulted in significant changes in the expression of many genes associated with angiogenesis and cancer pathways, further demonstrating the important roles of ZKSCAN3 in cancer progression. Although we know that ZKSCAN3 is involved in the progression of many cancers, the mechanisms by which it affects cancer progression are still unknown or controversial, and therefore need further investigation [31,32]. The application of various omics may play an important role in elucidating this problem.

In addition to promoting cancer progression, ZKSCAN3 has also been reported to play an important role in inhibiting the transcription of autophagic and lysosomal genes, and cellular senescence [5,33]. Chauhan et al. first reported that ZKSCAN3 can function as a transcriptional repressor of autophagy; besides, they believe that the growth defect caused by ZKSCAN3 silencing is probably due to the increased autophagy that leads to cellular senescence [5]. However, Pan et al. generated *Zkscan3* KO mice using the CRISPR/Cas9 system and found that the *Zkscan3* KO did not alter the transcription of autophagic and lysosomal genes [6]. Therefore, the study in *Zkscan3* KO mice suggested that the lacking of ZKSCAN3 was unable to promote the transcription of autophagic and lysosomal genes. In addition, Hu et al. generated *ZKSCAN3* KO human mesenchymal stem cells (hMSCs) and found that the *ZKSCAN3* KO was also unable to promote the transcription of autophagic and lysosomal genes in hMSCs [33]. These results suggest that *ZKSCAN3* KO cannot promote the transcription of autophagic and lysosomal genes in either mice or hMSCs. However, the effect of shRNA-mediated silencing of ZKSCAN3 on autophagy and lysosomal biogenesis have been proved in several established transformed human tumor cell lines by Chauhan et al. [5]. Moreover, Pan et al. also confirmed these observations using similar strategies [6], suggesting that the silencing of ZKSCAN3, but not the *ZKSCAN3* KO, can indeed induce autophagy and increase lysosomal biogenesis.

It may be due to different gene interventions or different cell types that the results of *ZKSCAN3* KO and ZKSCAN3 knockdown are inconsistent. To further rule out whether the results were inconsistent due to different cell types, we generated *ZKSCAN3* KO HK-2 (non-cancer) cells and Hela (cancer) cells via the CRISPR/Cas9 system. The focus of this study is on cancer and non-cancer cells and the differences in *ZKSCAN3* KO in these two cell types. Our results support Pan and Hu’s conclusion that *ZKSCAN3* KO was unable to promote the transcription of many autophagic and lysosomal genes. Combining the results of other people’s research with our results, we suppose that the reason for the difference between ZKSCAN3 knockdown and *ZKSCAN3* KO is probably due to the different ways of gene intervention [34].

Autophagy and aging are closely related [35,36]. Chauhan et al. found that silencing ZKSCAN3 via lentiviral particles that encode small hairpin RNAs (shRNAs) promotes cellular senescence [5]. They believe that cellular senescence is probably due to increased autophagy caused by ZKSCAN3 silencing. Hu et al. found that although *ZKSCAN3* KO cannot promote the transcription of autophagic and lysosomal genes, ZKSCAN3 can function as an epigenetic modulator to maintain heterochromatin organization and thereby inhibit hMSC senescence [33]. In HK-2 and Hela cells, we found that *ZKSCAN3* KO had no significant effect on the expression of p16 or lamin B1, which is known as the maker of cellular senescence. This may be due to different cell types, or the different ways in which we knocked out ZKSCAN, we targeted exon 3 of ZKSCAN3, while Hu et al. targeted exon 1 of ZKSCAN3.

In conclusion, CRISPR/Cas9 mediated *ZKSCAN3* KO experiments and whole transcriptome sequencing in the two mutated types of cells showed that *ZKSCAN3* KO was unable to promote the transcription of many autophagic and lysosomal genes in either HK-2 cells or Hela cells. ZKSCAN3 may act differently in different cells; it may be involved in cancer progression and aging processes, but the mechanisms need further elucidation.

## 4. Materials and Methods

### 4.1. Materials and Agents

The primary antibody against glyceraldehyde-phosphate dehydrogenase (GAPDH) was obtained from absin (#Ab5830030, Shanghai, China). The primary antibody against ZKSCAN3 was purchased from Santa Cruz Biotechnology (Santa Cruz, CA, USA). The primary antibody against LC3-II/I was obtained from Sigma (L7543, Saint Louis, MI, USA). The primary antibody against p62 was purchased from Abcam (Ab56416, Cambridge, UK). The primary antibody against p16 was obtained from Abcam (Ab51243, Cambridge, UK). The primary antibody against lamin B1 was purchased from Abcam (Ab16048, Cambridge, UK).

### 4.2. Construction of sgRNA-Expressing Plasmids

In order to completely disable the function of the ZKSCAN3 gene, we designed gRNA to cause frameshift mutations in exon 3, thus mutating both isoforms encoded by ZKSCAN3. The specific method for sgRNA-expressing plasmids construction in this study is the same as described previously [37]: Step 1—Synthesis of 20nt-guide (containing restriction sites); in the second step, the 20nt-guide was linked to the plasmids (PX458) linearized by the restriction enzyme; the third step was to make sure that the 20nt-guide was correctly connected through Sanger sequencing. The constructed vector can be used to transfect cells and pick monoclones.

### 4.3. sgRNA-Expressing Plasmids Transfection and ZKSCAN3-KO Cell Lines Screen

The constructed sgRNA-expressing plasmids were transfected into HK-2 and Hela cells by liposome. 48 h after plasmid transfection, the adherent cells were digested into single cells, and single GFP expressing cells were selected under fluorescence microscope and expanded for culture. DNA, RNA and proteins were extracted from the cells, and the ZKSCAN3-KO cell line was determined by PCR, Sanger sequencing and WB.

### 4.4. Western Blot

Cellular proteins were extracted by RIPA lysis buffer (Beyotime Biotechnology, #P0013B, Shanghai, China). The extracted proteins were then quantified with the BCA protein quantitation kit, and an equivalent amount of 20 µg proteins were loaded into 10% SDS-polyacrylamide gel for electrophoresis. After the electrophoresis is completed, the protein was transferred to polyethylene fluoride (PVDF) (Millipore) by the transfer system. The PVDF membranes were thereafter blocked with 5% Bovine Serum Albumin (BSA) in Tris-buffered solution containing 1% tween 20 (TBST) for 1 h. Next, the PVDF membrane was washed with PBST, and the corresponding primary antibodies were used to incubate the PVDF membrane overnight at 4 °C refrigerated. Then, the PVDF membrane was incubated with the secondary antibodies at room temperature for 1 h. Finally, the membranes were incubated with ECL chemiluminescence solution for 30 s, and then visualized with the Azure Biosystems C500 near-infrared imaging system.

### 4.5. Total RNA Isolation and qRT-PCR Analysis

Total cellular RNA was extracted from cultured cells using RNAiso Plus (Takara, 9108, Japan) according to the manufacturer’s protocol. After that, the RNA concentration and purity were quantified using NanoDrop 2000 ultrafine spectrophotometer (Thermo Scientific, MA, USA). And then the genome DNA was eliminated with the RNase-free DNase H. The RNA was then reverse-transcribed with the 5× FastKing-RT SuperMix kit (TIANGEN, KR118–01, Beijing, China) according to the manufacturer’s protocols. The reverse-transcribed cDNA was used as a template for qPCR using the Genious 2× SYBR Green Fast qPCR Mix (ABclonal, RK21204) according to the manufacturer’s protocol. The LightCycler 480 II real-time fluorescence quantitative PCR instrument was used for PCR reaction. The primer sequences used for amplifying the target fragments and GAPDH as an internal control were listed in Table S1. The gene’s relative expression levels were calculated by the 2^–ΔΔCt^ (threshold cycle) method.

### 4.6. Transcriptome Sequencing and Data Processing

Transcriptome sequencing and data processing were performed by GENEWIZ Biotechnology (GENEWIZ, Jiangsu, China). Transcriptome-sequencing procedures include RNA extraction, RNA sample quality detection, library construction, library purification, library detection, library quantification, generation of sequencing clusters and computer sequencing. In order to ensure the accuracy and reliability of source data, we conducted strict quality control for each step of the experimental process. After passing the test, different libraries were mixed according to the requirements of effective concentration and target data volume and then sequenced by Illumina. Bcl2fastq (v2.17.1.14) was used for image Base Calling for preliminary quality analysis to obtain the original sequencing Data (Pass Filter Data), and the results were stored in the FASTQ file format. It contains sequencing sequence information and its corresponding sequencing quality information. The sequencing data quality was evaluated by FastQC (v0.10.1) software. Raw data was preprocessed using Cutadapt (version 1.9.1) software and low quality data was filtered to remove contamination and joint sequences. The filtered sequencing Clean Data was compared with the reference genome for analysis. Hisat2 (v2.0.1) software was used to compare short reads with the default parameters. Gene expression was calculated using Cuffdiff software (v2.2.1), which calculated gene expression using FPKM (fragments per kilo bases per million reads) [38]. Genetic difference analysis was performed using Cuffdiff (v2.2.1), which was based on a negative binomial distribution model. The detection results were screened according to the differential significance criteria (differential gene expression changes of more than 2 times and Q-value (fdr, padj < =0.05), and the downregulation of significant differential gene expression was counted. The GO enrichment analysis method is GOseq [39], which is based on the Wallenius non-central hyper-geometric distribution. Compared with ordinary Hyper-geometric distribution, this distribution is characterized by the difference between the probability of drawing individuals from a certain class and probability of drawing individuals from outside a certain class. Such difference in probability is obtained by estimating the preference of gene length. Thus, the probability of GO term enrichment by differential genes can be calculated more accurately. The screening criterion for significant enrichment was: over_represented_*p* value ≤ 0.05. Pathway significance enrichment analysis using the KEGG pathway as a unit, hypergeometric test was applied to find out the pathway that showed significant enrichment in differentially expressed genes compared with the whole genome background. The screening criteria for significant enrichment was Q-value < 0.05. The differentially expressed genes are listed in Supplementary Table S3.

### 4.7. Statistical Analysis

All the results in this paper were presented as mean ± standard deviation (SD) and the statistical analysis was performed with the GraphPad Prism software, version 8.0.2. The two groups were compared by using Student’s *t*-test and *p* < 0.05 (*) and *p* < 0.01 (**) were considered significant and very significant, respectively.

## 5. Conclusions

*ZKSCAN3* KO was unable to promote the transcription of many autophagic and lysosomal genes in either HK-2 cells or Hela cells.

## Data Availability

The RNA-Seq data presented in this study are openly available in [NCBI GEO DataSets] at [https://www.ncbi.nlm.nih.gov/geo/query/acc.cgi?acc = GSE218727], accessed on 31 January 2023, reference number [GSE218727]. All data utilized in this study are included in this article, and all data supporting the findings of this study are available on reasonable request from the corresponding author (J.-X.T.).

## Supplementary Materials

The supporting information can be downloaded at: https://www.mdpi.com/article/10.3390/ijms24097786/s1.
