# Supplementary material for: Effect of Lacking ZKSCAN3 on Autophagy, Lysosomal Biogenesis and Senescence
Source: Int J Mol Sci. 2023 Apr 24;24(9):7786. doi: 10.3390/ijms24097786 (PMC10178544; doi:10.3390/ijms24097786)
Supplement: Supplementary file 1 [file ijms-24-07786-s001.zip › Figure S1 S2 Table S1.pdf]

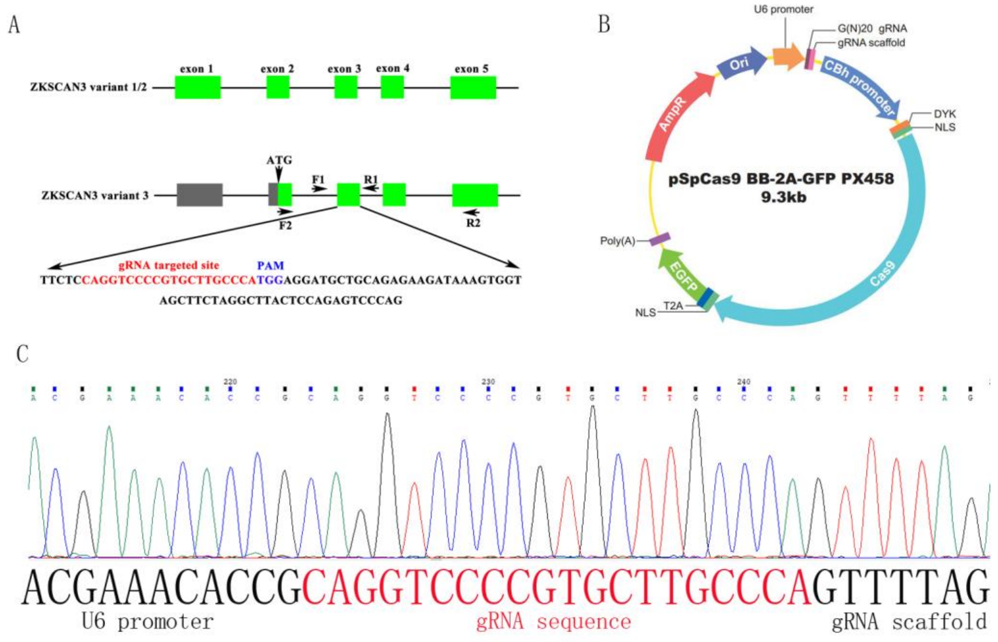

Figure S1 Construction of plasmid vector for ZKSCAN3 gene knockout. (A) Location of selected gRNA targeting sites on the ZKSCAN3 gene. The gRNA target site is located on exon 3 of the ZKSCAN3 gene and can target three transcripts of ZKSCAN3. (B) The plasmid vector eSpCas9-2A-GFP (PX458) was used. The vector contains Cas9 protein coding sequence and GFP fluorescent protein coding sequence. It also contains the U6 promoter sequence, after which the gRNA sequence is inserted. (C) Sequencing was used to determine whether the constructed plasmid vector was correct. By comparison, we found that the constructed carrier was exactly right.

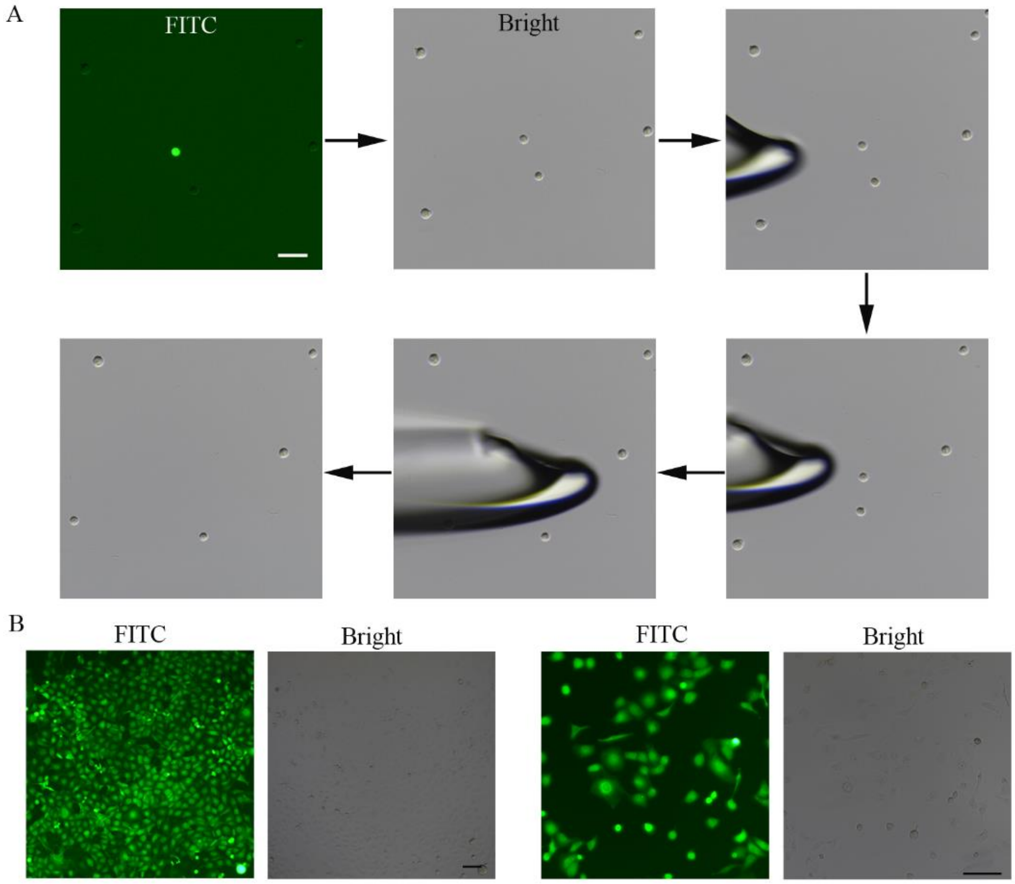

Figure S2 Single cell extraction by capillary glass straw. (A) Green fluorescent protein (GFP) expressed cell was picked up selected via capillary glass straws under an inverted fluorescence microscope and cultured in a 24-well plate to obtain monoclonal cell line. (B) Monoclonal GFP expressed cells after expanded culture. Bar=50 μ m.

Table S1. Primers for qPCR in this study.

Gene Name	Forward primer (5'-3')	Reverse primer (5'-3')	Gene ID
ATP6V1H	CAGAAGTTCGTGCAAACAAAGTC	TCAGGGCTTCGTTTCATTTCAA	51606
CTSA	GTCGCCCAGAGCAATTTTGAG	TCTCCCCGGTCAGGAAAAGTT	5476
CTSB	ACAACGTGGACATGAGCTACT	TCGGTAAACATAACTCTCTGGGG	1508
CTSD	TGCTCAAGAACTACATGGACGC	CGAAGACGACTGTGAAGCACT	1509
CTSF	AGCCCAAGTCAGCCTTCAC	CGCACCATGTTATTGACAAAGAC	8722
GALNS	GTGACCTCGGGGTGTATGGA	AAGCCATTGCGGATGGGTAG	2588
GBA	CATCCGCACCTACACCTATGC	TGAGCTTGGTATCTTCCTCTGG	2629
GLA	CTGAGGAACCCAGAACTACATCT	GGTAGGCGTCCTTGCCAAT	2717
NAGLU	ACCGCTATTACCAGAATGTGTG	CCATCCAGTCTATCTCTCGCTC	4669
NEU1	GGAGGCTGTAGGGTTTGGG	CACCAGACCGAAG TCGTTCT	4758
PSAP	CCCGGTCCTTGGACTGAAAG	TATGTCGCAGGGAAGGGATTT	5660
SCPEP1	CTGAACGCAGGAGCTGTCATT	CCTTGCGGACCGTCACATAAT	59342
SGSH	ACGGAGGCTTTGAGAGTGG	GCATTGCGAAAGAGGAGGCT	6448
TPP1	CCTCCACACGGTGCAAAAATG	CTCTGCTTGTCGGATGCTCAG	1200
HEXA	ACGTCCTTTACCCGAACAACT	CGAAAAGCAGGTCACGATAGC	3073
LAMP1	TCTCAGTGAACTACGACACCA	AGTGTATGTCCTCTTCCAAAAGC	3916

GAPDH	GGAGCGAGATCCCTCCAAAAT	GGCTGTTGTCATACTTCTCATGG	2597

Table S2. List of significantly differentially expressed genes in HK-2 cells.

Table S3. List of significantly differentially expressed genes in Hela cells.
